# Thymol-Decorated Gold Nanoparticles for Curing Clinical Infections Caused by Bacteria Resistant to Last-Resort Antibiotics

**DOI:** 10.1128/msphere.00549-22

**Published:** 2023-04-05

**Authors:** Zeyu Huang, Xiaotuan Zhang, Zhuocheng Yao, Yijia Han, Jianzhong Ye, Yi Zhang, Lijiang Chen, Mo Shen, Tieli Zhou

**Affiliations:** a Key Laboratory of Clinical Laboratory Diagnosis and Translational Research of Zhejiang Province, The First Affiliated Hospital of Wenzhou Medical University, Wenzhou, China; b School of Laboratory Medicine and Life Science, Wenzhou Medical University, Wenzhou, China; University of Rochester

**Keywords:** gold nanoparticles, thymol, last-resort antibiotic, multidrug resistance, antibacterial therapy, Gram-negative bacteria

## Abstract

Multidrug-resistant bacteria pose a tremendous challenge to public health worldwide. Many bacteria resistant to last-resort antibiotics due to antibiotic misuse have been recently reported, which may give rise to serious infections without effective treatment. Therefore, it is imperative to develop novel antimicrobial strategies. Natural phenols are known to increase bacterial membrane permeability and are potential candidates for the development of new antimicrobial agents. In this study, gold nanoparticles (Au NPs) carrying natural phenols were synthesized to combat bacteria resistant to last-resort antibiotics. Transmission electron microscopy, dynamic light scattering, zeta potential, and UV-visible spectra were used to characterize the synthesized Au NPs, which showed good monodispersity and uniform particle size. Evaluation of antibacterial activity using the broth microdilution method revealed that thymol-decorated gold nanoparticles (Thymol_Au NPs) had a broad antibacterial spectrum and higher bactericidal effects than last-resort antibiotics against last-resort-antibiotic-resistant bacteria. Considering the underlying antibacterial mechanism, the results showed that Thymol_Au NPs destroyed bacterial cell membranes. Further, Thymol_Au NPs were effective in treating mouse abdominal infections and exhibited acceptable biocompatibility without any significant toxicity in cell viability and histopathological assays, respectively, at most bactericidal concentrations. However, attention should be paid to changes in white blood cells, reticulocyte percentages, and superoxide dismutase activity during Thymol_Au NP treatment. In conclusion, Thymol_Au NPs have the potential for treating clinical infections caused by bacteria resistant to last-resort antibiotics.

**IMPORTANCE** Excessive use of antibiotics can lead to bacterial resistance and the development of multidrug-resistant bacteria. Antibiotic misuse can also promote resistance against last-resort antibiotics. It is thus crucial to develop alternatives to antibiotics to retard the development of multidrug resistance. In recent years, the use of several nanodosage forms of antibacterial drugs has been investigated. These agents kill bacteria through a variety of mechanisms and avoid the problem of resistance. Among them, Au NPs, which are safer to use for medical applications than other metal nanoparticles, have attracted interest as potential antibacterial agents. To combat bacterial resistance to last-resort antibiotics and mitigate the problem of antimicrobial resistance, it is important and meaningful to develop antimicrobial agents based on Au NPs.

## INTRODUCTION

The development of multidrug-resistant (MDR) bacteria is a major concern for public health worldwide (1) as it has undermined the efficacy of antibiotics used to treat infectious diseases (2) and is estimated to lead to widespread pan-drug resistance within the next 20 years (3). Antibiotics such as tigecycline (TGC) and colistin (COL) are used as a last resort for the treatment of diseases caused by MDR bacteria. Since their recent reinstallation in clinical practice, TGC- and COL-resistant microbial isolates have already been reported (4, 5). Ceftazidime-avibactam (CZA), a cephalosporin–β-lactamase inhibitor combination with a broad antibacterial spectrum, was introduced in 2015 and is regarded as a last-resort antibiotic (6). Several studies have reported the isolation of CZA-resistant strains in patients without prior treatment or following short exposure to CZA (7). Thus, the identification and development of new antimicrobial agents that are effective against bacteria resistant to last-resort antibiotics are critical.

Researchers have generally preferred the use of combination drug therapy to combat MDR bacteria as this can achieve better therapeutic effects (8). The drug combination can function synergistically by acting on different bacterial targets or pathways (9, 10). Natural phenols are classified as natural organic compounds that contain one or more phenolic groups in their structure (11). Natural phenols can not only inactivate surface-exposed adhesins, cell envelope transport proteins, and membrane-bound enzymes on the bacterial surface but also cause alterations in membrane permeability and metabolic pathways through the dysregulation of intracellular enzymes (12). Membrane permeability is a key factor in the design of novel antimicrobial strategies (13). As natural phenols can increase the permeability of bacterial cell membranes, they have been found to have antimicrobial activity either alone or in combination with traditional antibiotics (14–17). Our previous studies showed that four types of natural phenols, namely, thymol (18), plumbagin (19), naringenin (20), and kaempferol (21), had synergistic antimicrobial effects on colistin-resistant (COL-R) Gram-negative bacteria when used in combination with COL. These results suggested that natural phenols exerted synergistic antimicrobial activities most likely by increasing bacterial cell membrane sensitivity. However, natural phenols have poor water solubility and require high doses, which limits their clinical use. As hydrophobic drugs, natural phenols are commonly solubilized in dimethyl sulfoxide, which is toxic to human cells (22). Further, when used in combination with other drugs, natural phenols are used at higher concentrations than traditional antibiotics. Therefore, it is essential to identify novel approaches to enhance the antimicrobial activity of natural phenols by increasing cell membrane permeability.

The application of nanotechnology may offer solutions to these issues, and several nanodosage forms of antibacterial medications have been investigated (23). Inorganic nanoparticles with antimicrobial properties and wide activity ranges, such as gold nanoparticles (Au NPs), silver nanoparticles, zinc oxide nanoparticles, and titanium dioxide nanoparticles, have shown remarkable results (24–26). In particular, gold nanostructures have shown excellent antibacterial potential against MDR bacteria (27). Many forms of Au NPs have been reported, including hollow Au nanospheres, Au nanorods, Au nanotubes, and Au nanocages (28, 29). Natural phenols are suitable for application in Au NP synthesis due to the chemical structure of the phenolic hydroxyl group. Therefore, the potential antibacterial activity of these natural phenols against Gram-negative bacteria resistant to last-resort antibiotics was explored through their synthesis into Au NPs.

In this study, natural phenols were successfully synthesized into Au NPs and investigated for their antibacterial activities and mechanisms, as well as biosafety. Our study showed that thymol-decorated gold nanoparticles (Thymol_Au NPs) had good antimicrobial activity against clinical Gram-negative bacteria resistant to last-resort antibiotics *in vitro* and *in vivo*. The bactericidal effect was achieved by altering the permeability of the bacterial cell membrane. Our work thus demonstrated a safe and effective antimicrobial strategy.

## RESULTS AND DISCUSSION

### Characterization of different Au NPs.

**FIG 1:**
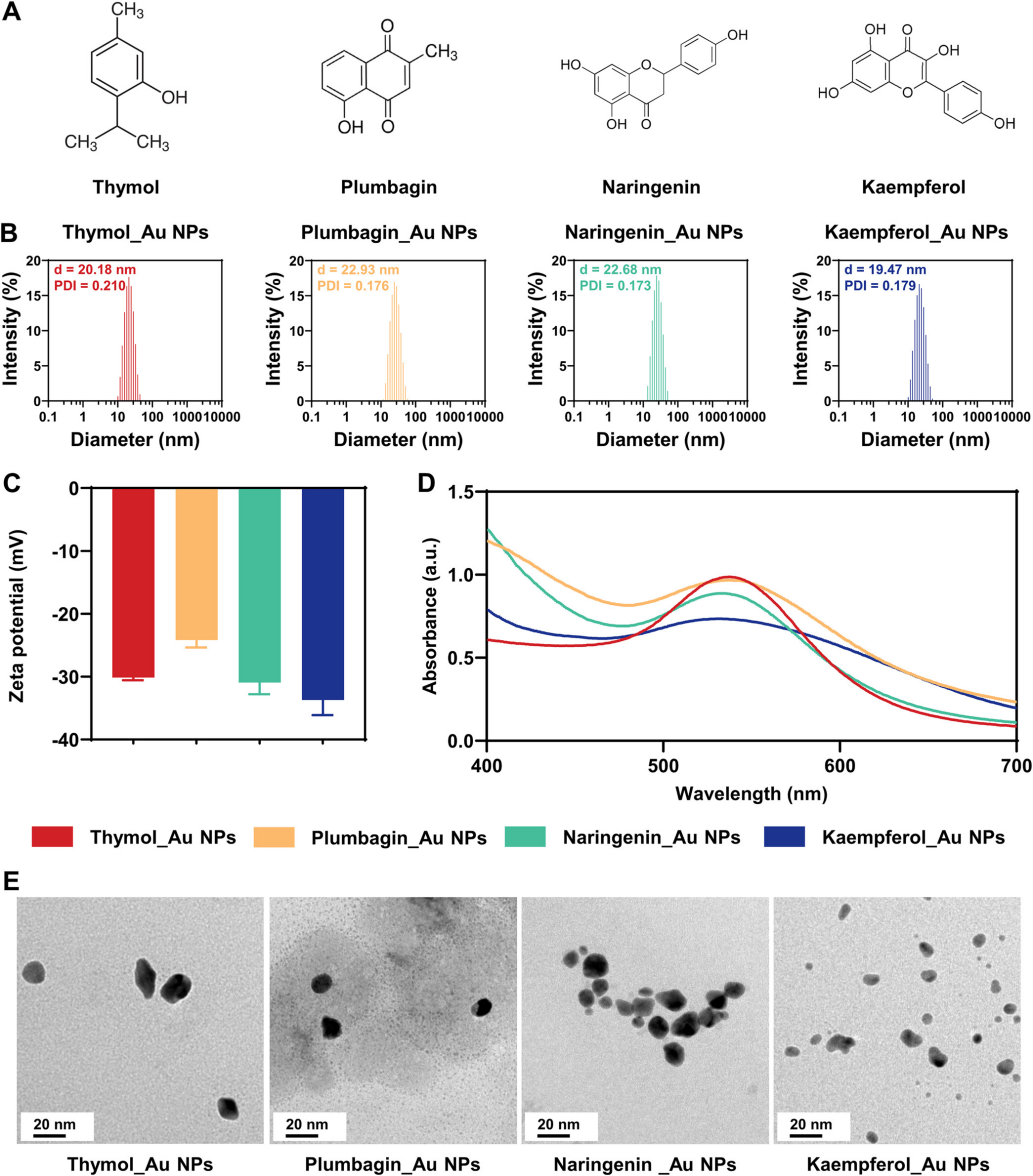
Characterization of different Au NPs. (A) Molecular formulae of thymol, plumbagin, naringenin, and kaempferol. (B) Particle size distributions of Thymol_Au NPs, Plumbagin_Au NPs, Naringenin_Au NPs, and Kaempferol_Au NPs. (C) Zeta potentials of Thymol_Au NPs, Plumbagin_Au NPs, Naringenin_Au NPs, and Kaempferol_Au NPs. (D) UV-visible spectra of Thymol_Au NPs, Plumbagin_Au NPs, Naringenin_Au NPs, and Kaempferol_Au NPs. (E) TEM images of Thymol_Au NPs, Plumbagin_Au NPs, Naringenin_Au NPs, and Kaempferol_Au NPs.

The natural phenols used in this study had one or three phenolic hydroxyl groups that exhibit reducibility (Fig. 1A). Nanoparticles were synthesized by reducing chloroauric acid (HAuCl_4_) with an excess of natural phenols. Au^3+^ could be reduced to Au^0^ in chloroauric acid to generate the corresponding Au NPs through the Au-O bond, due to the reducibility of the natural phenols. Unreacted chemicals were removed by dialysis. The synthesized Au NPs were stable in aqueous solution and exhibited good uniformity, indicating that the incorporation of the natural phenols into Au NPs could improve their water solubility. As determined by a nanoparticle size and zeta potential analyzer (dynamic light scattering [DLS]) (Fig. 1B), the average sizes of the Thymol_Au NPs, Plumbagin_Au NPs, Naringenin_Au NPs, and Kaempferol_Au NPs were 20.18, 22.93, 22.68, and 19.47 nm, respectively, and their polymer dispersity indices (PDIs) were 0.210, 0.176, 0.173, and 0.179, respectively. As shown in Fig. 1C, negative zeta potential values of −30.13 ± 0.45 mV, −24.17 ± 1.19 mV, −33.70 ± 2.41 mV, and −30.93 ± 1.85 mV were obtained for Thymol_Au NPs, Plumbagin_Au NPs, Naringenin_Au NPs, and Kaempferol_Au NPs, respectively, suggesting their stability. Their adsorption peaks ranged between wavelengths of 500 and 600 nm, which corresponded to the characteristic surface plasmon resonance of Au NPs (Fig. 1D). As shown in Fig. 1E, transmission electron microscopy (TEM) images showed that these four types of Au NPs were well dispersed and had granular structures. Their average size (about 10 nm) in TEM was generally smaller than that in DLS, which could be explained by the fact that TEM measurements were taken in a dried state, whereas DLS measurements were taken in a hydrated state (30). The content of elemental gold was measured with an inductively coupled plasma optical emission spectrometer (ICP-OES) for subsequent research.

### Evaluation of *in vitro* antibacterial activity and the underlying mechanism.

**FIG 2:**
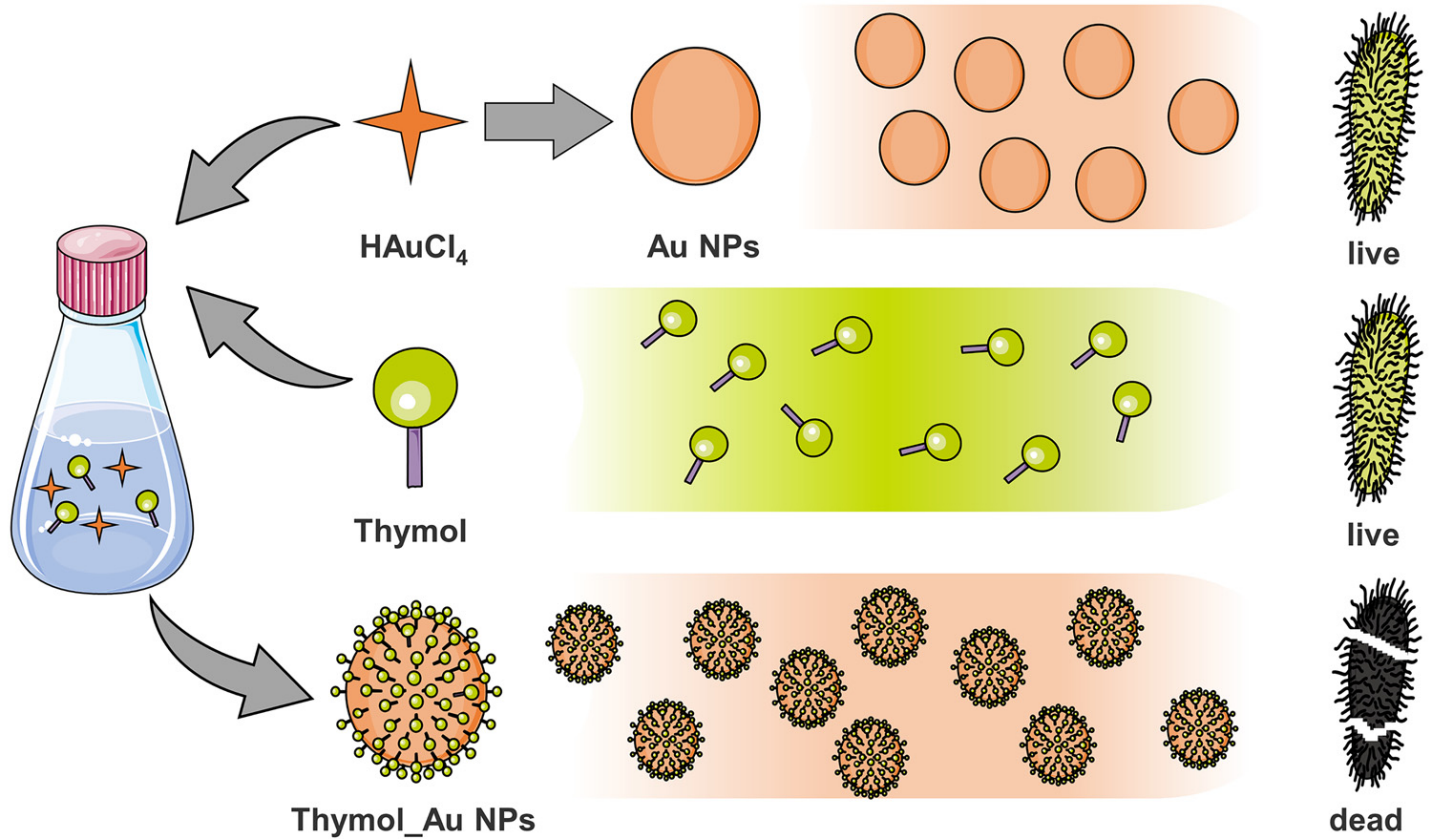
Antibacterial schematic diagram of thymol, unmodified Au NPs, and Thymol_Au NPs.

Nine TGC-resistant (TGC-R), 10 COL-R, and 12 CZA-resistant (CZA-R) Gram-negative bacterial strains were chosen from the clinic. Tables S1 to S5 in the supplemental material show the susceptibilities of these bacteria to the majority of common antibiotics used in clinical treatment. Overall, 87.1% (27/31) of the strains had MDR phenotypes, while 77.4% (24/31) of the strains were resistant to imipenem, and 77.2% (23/31) were resistant to ciprofloxacin. Thus, the strains used in this study exhibited severe antimicrobial resistance. As shown in Table 1, the TGC MIC values for the TGC-R strains ranged from 16 to 32 μg/mL while the COL MIC values for the COL-R strains ranged between 8 and 256 μg/mL, and those for the CZA-R strains were ≥512/4 μg/mL. The Naringenin_Au NP and Kaempferol_Au NP MIC values for all the strains were ≥128 μg/mL, indicating that these agents lacked significant antibacterial activity. Plumbagin_Au NPs, however, showed antibacterial activity against most of the strains tested, apart from several TGC-R and CZA-R strains. Surprisingly, Thymol_Au NPs showed excellent antibacterial activity against all strains, with MIC values ranging from 8 to 16 μg/mL, except for COL-R Klebsiella pneumoniae FK1913, CZA-R Enterobacter cloacae CG1257, and K. pneumoniae FK9283, where the MIC values were 64, 32, and 32 μg/mL, respectively. Thymol_Au NPs exerted a better bactericidal effect than other Au NPs possibly due to the single benzene ring and three methyl groups of thymol, resulting in higher hydrophobicity than that of other Au NPs. High hydrophobicity leads to higher affinity for the lipid components of the cell membrane (31), which might play a crucial role in mediating bactericidal effects. The original concentration of thymol in the Thymol_Au NP solution was 751.1 μg/mL before dialysis. After dialysis and dilution, the concentration of thymol in the Thymol_Au NP solution at most bactericidal dosages was less than 23.19 μg/mL, which was lower than the concentrations used in checkerboard assays where most synergistic bactericidal activities were observed at 64- and 32-μg/mL thymol concentrations in our previous study (18). Therefore, incorporating thymol into Au NPs could lower its effective concentration. The MIC values of the uncomplexed thymol and the unmodified Au NPs were also measured. TGC-R K. pneumoniae FK6768, COL-R Pseudomonas aeruginosa TL3008, and CZA-R P. aeruginosa TL3077 were randomly selected as experimental strains. The results showed that the thymol MIC values for these strains were ≥256 μg/mL while the unmodified Au NP MIC values were ≥128 μg/mL, indicating no significant antibacterial activity (Fig. 2 and Table 2). Considering that Thymol_Au NPs had a good bactericidal effect on most bacteria and were more active than last-resort antibiotics, we chose them for the subsequent determinations of antibacterial activity, antibacterial mechanism, and biosafety.

**TABLE 1:** Antimicrobial activities of last-resort antibiotics and different gold nanoparticles against the 31 clinical isolates used in this study

Type of antibiotic resistance	Species	Strain	MIC value (μg/mL)^*a*^						
			TGC	COL	CZA	Thymol_Au NPs	Plumbagin_Au NPs	Naringenin _Au NPs	Kaempferol_Au NPs
TGC resistance	A. baumannii	BM7438	32			16	≥128	≥128	≥128
		BM7404	32			16	64	≥128	≥128
		BM7481	32			16	≥128	≥128	≥128
		BM7338	32			16	64	≥128	≥128
		BM7594	32			8	≥128	≥128	≥128
		BM5333	32			8	64	≥128	≥128
		BM7580	16			8	≥128	≥128	≥128
		BM7389	16			8	≥128	≥128	≥128
	K. pneumoniae	FK6768	16			8	64	≥128	≥128
COL resistance	A. baumannii	BM1595		32		16	32	≥128	≥128
		BM2431		32		16	32	≥128	≥128
	E. coli	DC5286		16		8	8	≥128	≥128
		DC3599		8		8	16	≥128	≥128
	E. cloacae	CG648		256		16	32	≥128	≥128
		CG741		128		16	16	≥128	≥128
	K. pneumoniae	FK1913		128		64	32	≥128	≥128
		FK3810		128		16	32	≥128	≥128
	P. aeruginosa	TL3086		32		8	16	≥128	≥128
		TL3008		16		8	64	≥128	≥128
CZA resistance	E. coli	DC7914			≥512/4	16	8	≥128	≥128
		DC7956			≥512/4	16	8	≥128	≥128
		DC8439			≥512/4	16	16	≥128	≥128
	E. cloacae	CG1257			≥512/4	32	32	≥128	≥128
		CG1400			≥512/4	16	≥128	≥128	≥128
		CG1593			≥512/4	16	≥128	≥128	≥128
	K. pneumoniae	FK8966			≥512/4	16	≥128	≥128	≥128
		FK9102			≥512/4	16	≥128	≥128	≥128
		FK9283			≥512/4	32	32	≥128	≥128
	P. aeruginosa	TL2918			≥512/4	16	64	≥128	≥128
		TL2964			≥512/4	16	64	≥128	≥128
		TL3077			≥512/4	16	64	≥128	≥128

a TGC, tigecycline; COL, colistin; CZA, ceftazidime-avibactam; Thymol_Au NPs, thymol-decorated gold nanoparticles; Plumbagin_Au NPs, plumbagin-decorated gold nanoparticles; Naringenin_Au NPs, naringenin-decorated gold nanoparticles; Kaempferol_Au NPs, kaempferol-decorated gold nanoparticles.

**TABLE 2:** Antimicrobial activities of thymol, Au NPs alone, and Thymol_Au NPs against the FK6768, TL3008, and TL3077 strains

Strain	MIC (μg/mL)		
	Thymol	Au NPs	Thymol_Au NPs
FK6768	≥256	≥128	8
TL3008	≥256	≥128	16
TL3077	≥256	≥128	16

10.1128/msphere.00549-22.4 TABLE S1 MIC values against the five clinical E. coli isolates used. Abbreviations: AMP, ampicillin; SAM, ampicillin-sulbactam; TZP, piperacillin-tazobactam; CFZ, cefazolin; CTT, cefotetan; ATM, aztreonam; CRO, ceftriaxone; FEP, cefepime; ETP, ertapenem; IPM, imipenem; CIP, ciprofloxacin; LVX, levofloxacin; GEN, gentamicin; TOB, tobramycin; AMK, amikacin; NIT, nitrofurantoin; R, resistance. MDR strains are marked in red. Download Table S1, DOCX file, 0.02 MB. Copyright © 2023 Huang et al. 2023 Huang et al. https://creativecommons.org/licenses/by/4.0/ This content is distributed under the terms of the Creative Commons Attribution 4.0 International license.

**FIG 3:**
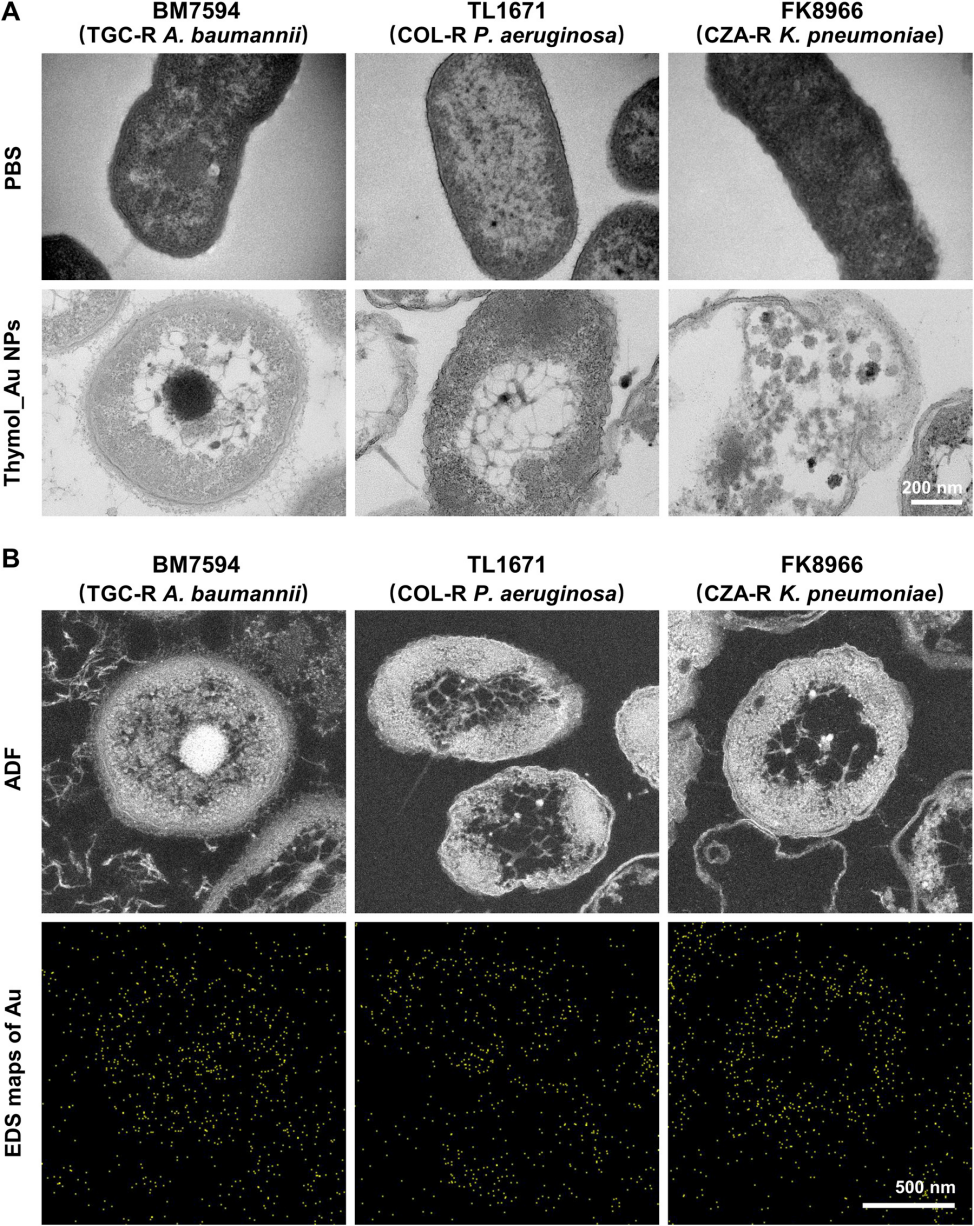
TEM/EDS analysis of Thymol_Au NP-treated bacteria. (A) The morphology of bacteria exposed to Thymol_Au NPs. (B) Annular dark field (ADF) and corresponding overlaid EDS maps of Au.

We investigated the bactericidal effects of Thymol_Au NPs by analyzing morphological changes in the bacteria by TEM after treatment with Thymol_Au NPs (Fig. 3A). The concentrations of Thymol_Au NPs used for TEM were derived from the results of the antimicrobial susceptibility assay. The TGC-R Acinetobacter baumannii BM7594, COL-R P. aeruginosa TL1671, and CZA-R K. pneumoniae FK8966 were randomly chosen as experimental strains. In the BM7594 and TL1671 strains, the bacterial wall structure became blurred, loose, and distorted. In the FK8966 strain, the cell was completely disintegrated. To evaluate the internalization of Thymol_Au NPs in the experimental strains, TEM/energy dispersive spectrometry (EDS) was performed to confirm the position of Thymol_Au NPs inside the bacteria (Fig. 3B). The results indicated that several Thymol_Au NPs could be detected inside the bacterial cells.

**FIG 4:**
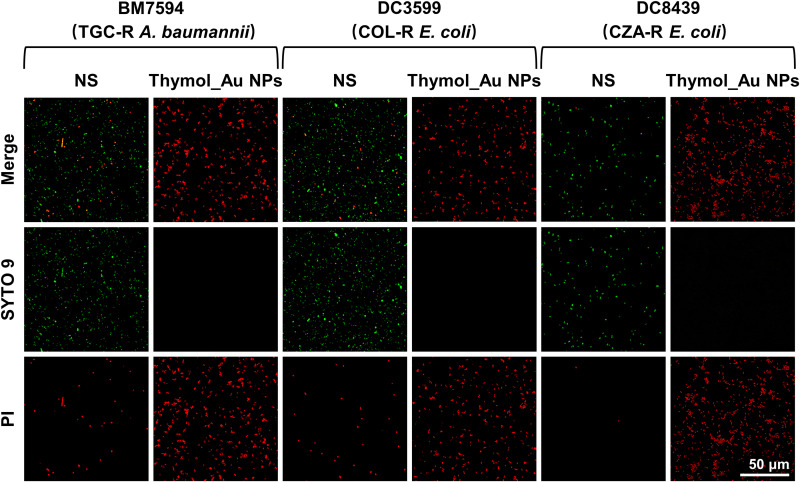
SYTO 9/PI staining to evaluate permeability of the bacterial cell membrane after Thymol_Au NP treatment.

To further demonstrate that changes in the membrane permeability were responsible for the entry of Thymol_Au NPs into the bacterial cells, a live/dead viability assay was performed. The TGC-R A. baumannii BM7594, COL-R Escherichia coli DC3599, and CZA-R E. coli DC8439 were randomly selected as experimental strains, and the concentrations of Thymol_Au NPs depended on the results of the antimicrobial susceptibility assay. In this experiment, bacterial cells with increased membrane permeability were visualized with propidium iodide (PI), which emitted red fluorescence, while normal bacteria were visualized with the green fluorescent probe SYTO 9. As shown in Fig. 4, weak red fluorescence was visible in the normal saline (NS) group, indicating the presence of normal cell membrane permeability after incubation for 4 h at 37°C without any treatment. In contrast, the group treated with Thymol_Au NPs showed intense red fluorescence with no green fluorescence signals, suggesting that the majority of the bacteria had compromised cell membrane permeability.

**FIG 5:**
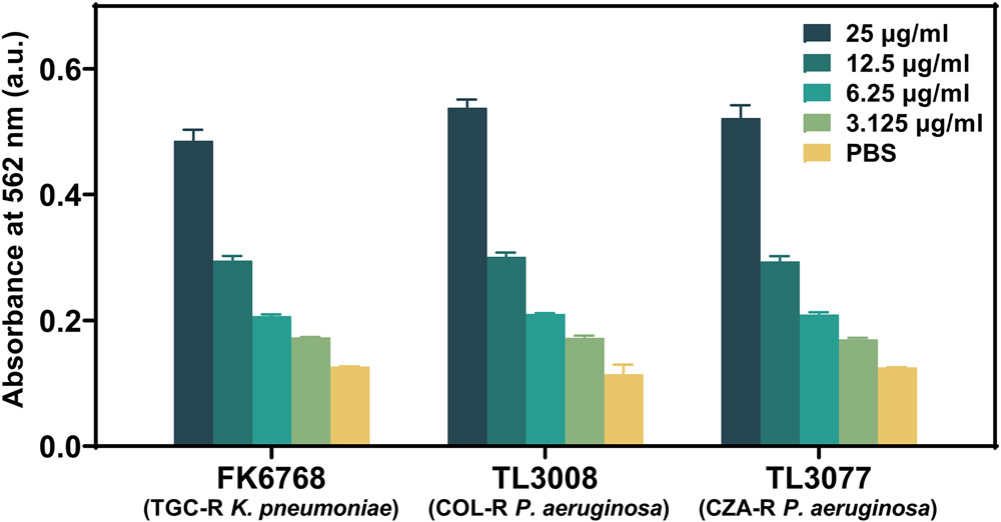
Intracellular protein leakage in bacteria after 6 h of treatment with PBS or Thymol_Au NPs.

To confirm these findings, an intracellular protein leakage assay (Fig. 5) was performed using the TGC-R K. pneumoniae FK6768, COL-R P. aeruginosa TL3008, and CZA-R P. aeruginosa TL3077 as randomly selected experimental strains. Thymol_Au NPs at subinhibitory concentrations were found to promote the leakage of intracellular proteins in a concentration-dependent manner, which indicated the gradual deterioration of bacterial cell membrane integrity.

Based on the data above and our previous study results (18), we speculate that thymol increased the permeability of the bacterial cell membrane, thus enhancing the entry of the nanomaterials into the cell and the consequent bactericidal effect. The TEM results, however, showed that the cell membranes of BM7594 and TL1671 were not completely disintegrated, which is suggestive of alternative mechanisms such as disturbance in metabolism, transcription, and translation.

**FIG 6:**
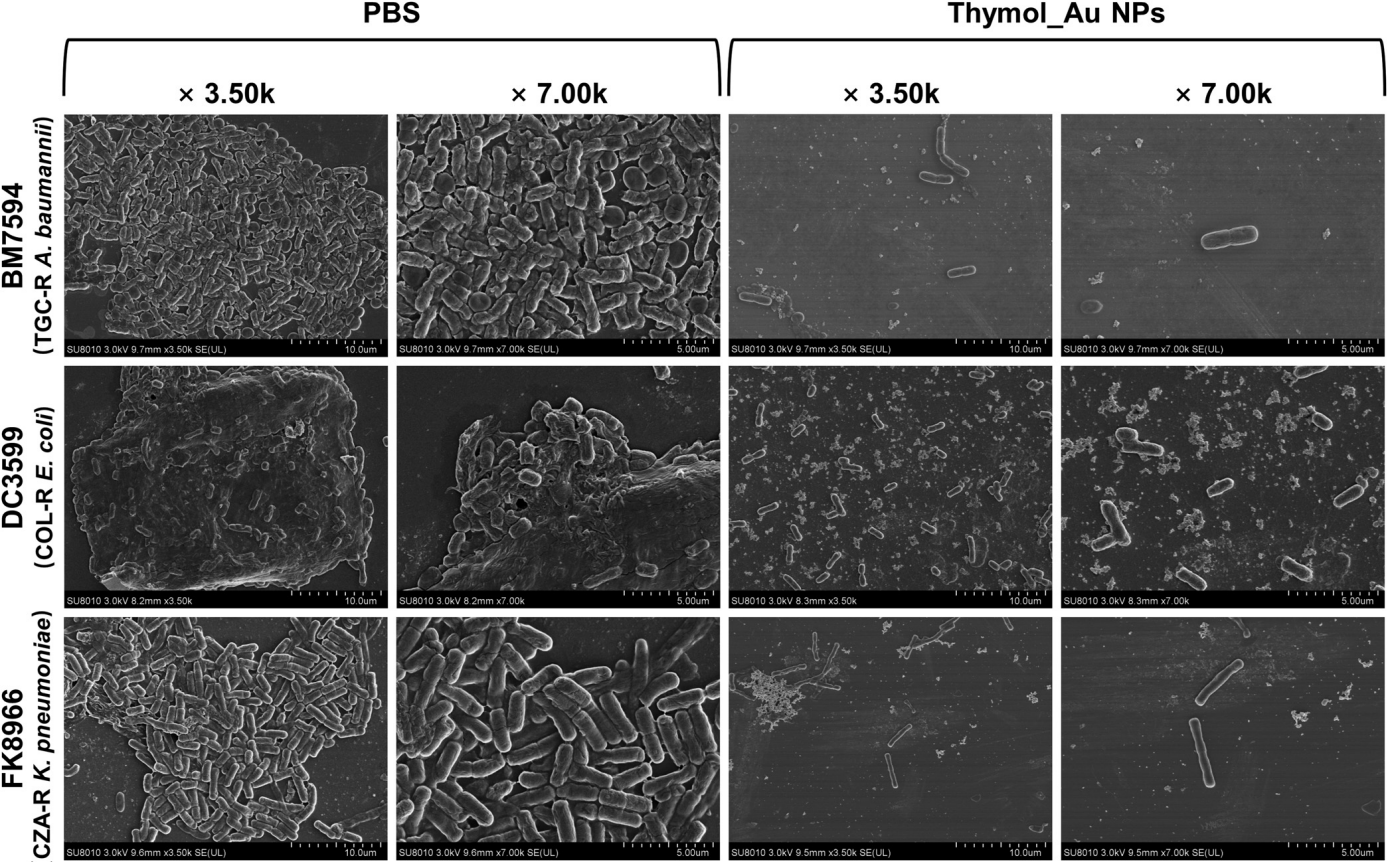
SEM image of Thymol_Au NP-treated bacteria.

Bacterial biofilm is a major cause of bacterial resistance to antibiotics and a key factor contributing to chronic infections and microbial survival under extreme conditions (32–34). To further characterize the inhibitory effect of Thymol_Au NPs on biofilms, scanning electron microscopy (SEM) was performed. The TGC-R A. baumannii BM7594, COL-R E. coli DC3599, and CZA-R K. pneumoniae FK8966 were randomly chosen as experimental strains. The concentrations of Thymol_Au NPs used depended on the results of the antimicrobial susceptibility assay. As shown in Fig. 6, the main field of view was completely covered by untreated bacterial cell biofilms in the NS group. However, Thymol_Au NPs induced a significant reduction in the bacterial quantity and density. K. pneumoniae FK8966 showed morphological changes, becoming elongated in the presence of Thymol_Au NPs.

### Antibacterial activity evaluation *in vivo*.

**FIG 7:**
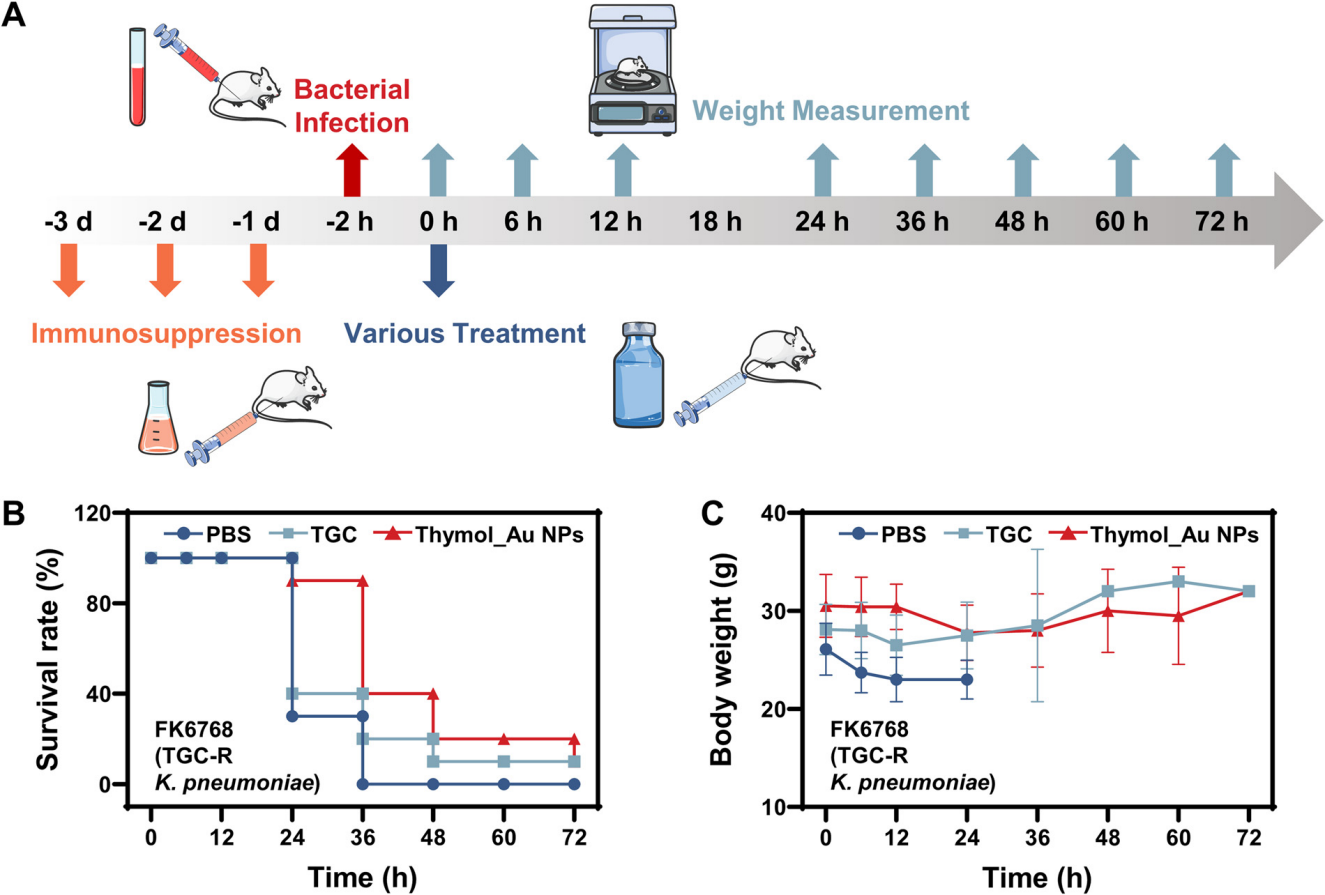
Single-dose treatment of intra-abdominally infected mice. (A) Schematic diagram of the mouse handling process. (B) Survival ratios of FK6768-infected mice treated with Thymol_Au NPs, TGC, or PBS. (C) Body weight analysis of infected mice treated with Thymol_Au NPs, TGC, or PBS.

We assessed the survival rates and body weights of mice at various time points to determine the therapeutic effect of Thymol_Au NPs *in vivo* (Fig. 7). An acute intra-abdominal infection mouse model was established after injection of the TGC-R K. pneumoniae FK6768. The schematic of the mouse experiment is shown in Fig. 7A. As shown in Fig. 7B, at the first 24 h, 30% of the mice treated with phosphate-buffered saline (PBS), 40% treated with TGC, and 90% of those treated with Thymol_Au NPs survived. However, at 36 h, 60% of the mice treated with Thymol_Au NPs died. Figure 7C indicates that the body weights of mice treated with PBS or TGC decreased at 12 h, which might be attributed to bacterial toxicity and ineffective treatment, while the body weights of the mice treated with Thymol_Au NPs showed no significant changes. The body weights of the mice treated with Thymol_Au NPs decreased at 24 h, which might explain the massive death at the 36-h time point. For the mice treated with PBS or TGC, there was no significant trend in the body weights between 24 and 72 h because most animals died and individual differences in the same group were unavoidable. Similarly, no trend was observed in the mice in the Thymol_Au NP treatment group between 36 and 72 h. Considering the deficient immune system of the mice and the high number of bacteria injected, we suspected that continuous treatment might be required to improve the survival rate.

**FIG 8:**
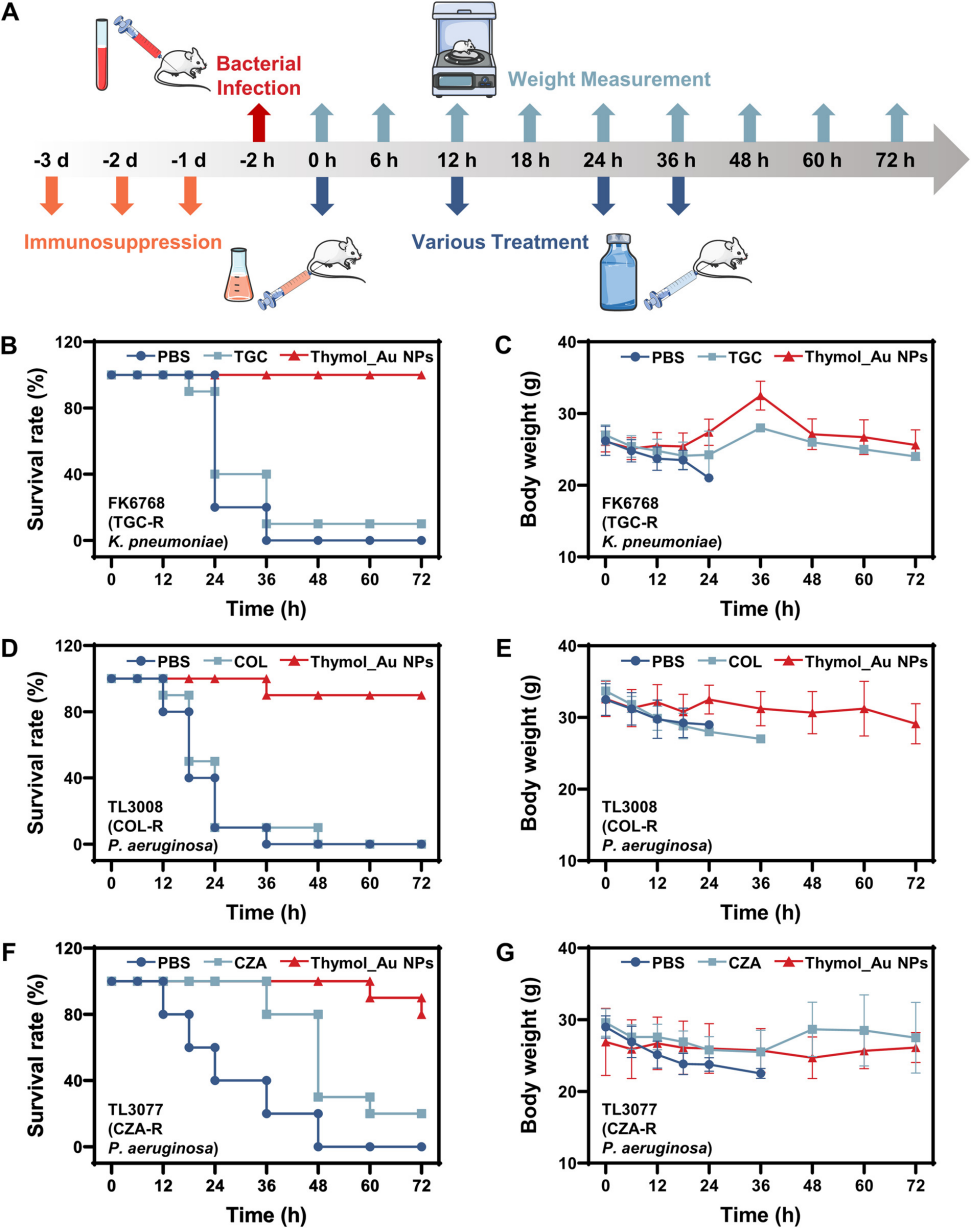
Continuous treatment of intra-abdominally infected mice. (A) Schematic diagram of the mouse handling process. (B) Survival ratios of FK6768-infected mice treated with Thymol_Au NPs, TGC, or PBS. (C) Body weight analysis of FK6768-infected mice treated with Thymol_Au NPs, TGC, or PBS. (D) Survival ratios of TL3008-infected mice treated with Thymol_Au NPs, COL, or PBS. (E) Body weight analysis of TL3008-infected mice treated with Thymol_Au NPs, COL, or PBS. (F) Survival ratios of TL3077-infected mice treated with Thymol_Au NPs, CZA, or PBS. (G) Body weight analysis of TL3077-infected mice treated with Thymol_Au NPs, CZA, or PBS.

We, therefore, constructed a continuous treatment model (Fig. 8). The TGC-R K. pneumoniae FK6768, COL-R P. aeruginosa TL3008, and CZA-R P. aeruginosa TL3077 were randomly selected as experimental strains. Figure 8A shows the details of the animal experiment. As shown in Fig. 8B, after 72 h, all the mice treated with PBS died while 10% of the mice treated with TGC survived and 100% of the mice treated with Thymol_Au NPs were alive. Thus, the body weights of the mice between 0 and 18 h decreased after treatment with PBS or TGC (Fig. 8C), given that most animals died. There was a steady rise in body weight between 0 and 36 h in the mice of the Thymol_Au NP treatment group, after which the body weights decreased dramatically to the level observed at 72 h. Figure 8D indicates that the mice treated with PBS or COL all died after 72 h but 90% of the mice treated with Thymol_Au NPs survived. The body weights of the mice treated with PBS or COL decreased between 0 h and 12 h (Fig. 8E) while no trend was observed in the weights of the mice treated with Thymol_Au NPs. As shown in Fig. 8F, after 72 h, all the mice treated with PBS died, 20% of the mice from the CZA treatment group survived, and 80% of the mice treated with Thymol_Au NPs survived. Figure 8G shows the steady weight loss observed in the mice treated with PBS. Considering that numerous mice treated with CZA died in the first 48 h, the body weights decreased between 0 and 36 h. No significant trend was observed in the body weights of the mice treated with Thymol_Au NPs. Figure S1 in the supplemental material shows the bacterial burden in the major organs of the mice from the continuous treatment experiment. Specifically, treatment with either PBS or the antibiotic resulted in a high bacterial burden in the major organs while Thymol_Au NP treatment significantly reduced the bacterial burden.

10.1128/msphere.00549-22.1 FIG S1 Bacterial burden of the major organs of mice from the continuous treatment model. (A) Bacterial burden of FK6768-infected mice treated with Thymol_Au NPs, TGC, or PBS. (B) Bacterial burden of TL3008-infected mice treated with Thymol_Au NPs, COL, or PBS. (C) Bacterial burden of TL3077-infected mice treated with Thymol_Au NPs, CZA, or PBS. Download FIG S1, TIF file, 0.6 MB. Copyright © 2023 Huang et al. 2023 Huang et al. https://creativecommons.org/licenses/by/4.0/ This content is distributed under the terms of the Creative Commons Attribution 4.0 International license.

### Biocompatibility assessment *in vitro*.

**FIG 9:**
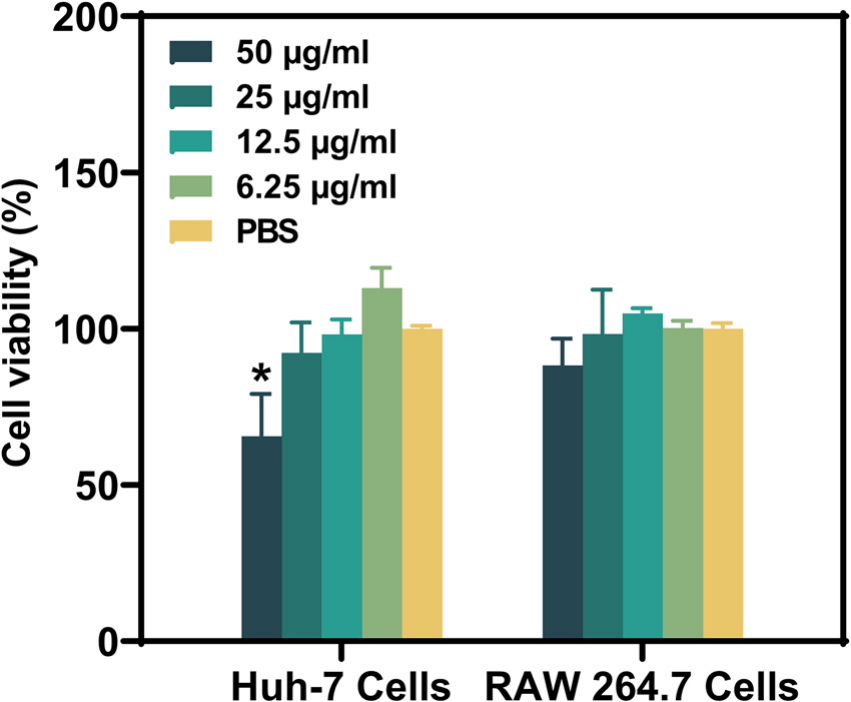
Cell viability of Huh-7 and RAW 264.7 cells after treatment with Thymol_Au NPs.

RAW 264.7 murine macrophage cells and Huh-7 liver cancer cells were used to assess cell viability. As shown in Fig. 9, treatment with Thymol_Au NPs resulted in no significant toxicity to Huh-7 and RAW 264.7 cells at concentrations ranging from 6.25 to 50 μg/mL, except at the 50-μg/mL concentration in Huh-7 cells, indicating its biosafety at most bactericidal concentrations.

### Biocompatibility assessment *in vivo*.

**FIG 10:**
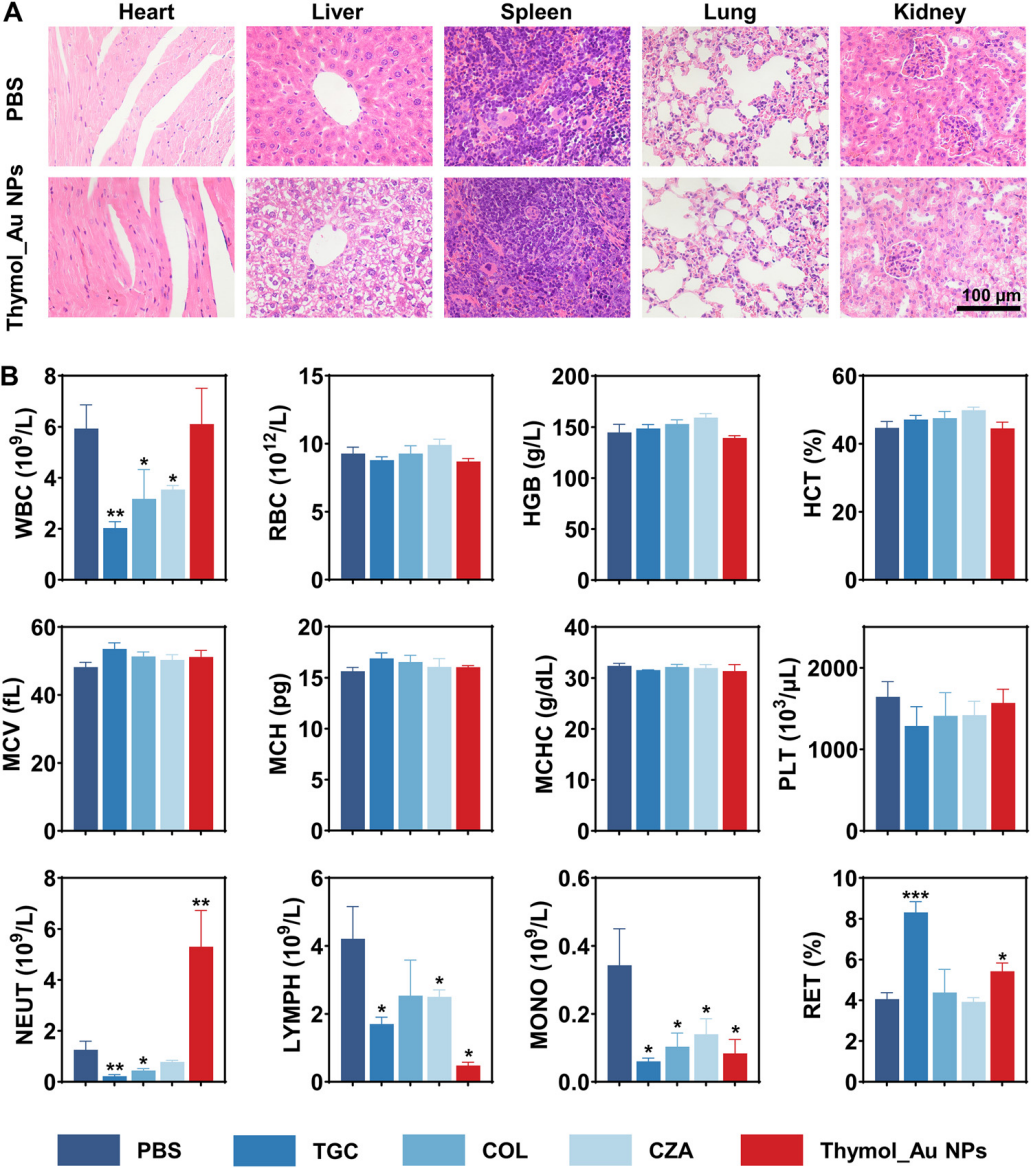
Biocompatibility of Thymol_Au NPs *in vivo*. (A) H&E-stained images of the main organs from mice after treatment with PBS or Thymol_Au NPs. (B) Major blood cell parameters of mice after treatment with PBS, TGC, COL, CZA, or Thymol_Au NPs. WBC, white blood cells; RBC, red blood cells; HGB, hemoglobin; HCT, hematocrit; MCV, mean corpuscular volume; MCH, mean corpuscular hemoglobin; MCHC, mean corpuscular hemoglobin concentration; PLT, platelets; NEUT, neutrophil count; LYMPH, lymphocyte count; MONO, monocyte count; RET (%), reticulocyte percentage.

Pathological sections from the organs of mice treated with PBS or Thymol_Au NPs were evaluated. As shown in Fig. 10A, no obvious histological damage was observed in the major organs. Routine blood tests were then performed in mice treated with PBS, TGC, COL, CZA, or Thymol_Au NPs. Figure 10B shows that the neutrophil count (NEUT), lymphocyte count (LYMPH), and monocyte count (MONO) in the TGC, COL, and CZA treatment groups were significantly lower than those in the PBS treatment group. The NEUT level in the Thymol_Au NP treatment group was significantly higher than that in the PBS treatment group. In terms of the reticulocyte percentage [RET (%)], TGC and Thymol_Au NP treatment significantly increased the levels of RET (%) compared to the PBS treatment. Given that white blood cell (WBC) and NEUT levels are important indicators of effective treatment, we measured these values during the study period. The timing of blood collection and the corresponding injection of Thymol_Au NPs are shown in Table S6. The results indicated that continuous administration of Thymol_Au NPs increased both the WBC and NEUT levels in mice (Fig. S2), which requires further investigation in subsequent clinical studies. In addition, the serum activity of superoxide dismutase (SOD) and the level of malondialdehyde (MDA) were examined, both of which are important indicators of oxidative stress. The results showed that there was no difference in MDA levels between the PBS-treated and Thymol_Au NP-treated mice, but there was a difference in SOD activity (Fig. S3), indicating that Thymol_Au NPs could eliminate oxygen free radicals from the body to a certain extent, thus protecting normal cell function. Thus, future studies should investigate the effects of Thymol_Au NPs on the immune, hematopoietic, and other functional systems.

10.1128/msphere.00549-22.2 FIG S2 Continuous detection of WBC and NEUT levels in Thymol_Au NP-treated mice. Download FIG S2, TIF file, 0.08 MB. Copyright © 2023 Huang et al. 2023 Huang et al. https://creativecommons.org/licenses/by/4.0/ This content is distributed under the terms of the Creative Commons Attribution 4.0 International license.

10.1128/msphere.00549-22.3 FIG S3 SOD (A) and MDA (B) levels in PBS-treated or Thymol_Au NP-treated mice. Download FIG S3, TIF file, 0.2 MB. Copyright © 2023 Huang et al. 2023 Huang et al. https://creativecommons.org/licenses/by/4.0/ This content is distributed under the terms of the Creative Commons Attribution 4.0 International license.

10.1128/msphere.00549-22.5 TABLE S2 MIC values against the 10 clinical A. baumannii isolates used. Abbreviations: SAM, ampicillin-sulbactam; CRO, ceftriaxone; FEP, cefepime; IPM, imipenem; CIP, ciprofloxacin; LVX, levofloxacin; R, resistance. MDR strains are marked in red. Download Table S2, DOCX file, 0.02 MB. Copyright © 2023 Huang et al. 2023 Huang et al. https://creativecommons.org/licenses/by/4.0/ This content is distributed under the terms of the Creative Commons Attribution 4.0 International license.

10.1128/msphere.00549-22.6 TABLE S3 MIC values against the six clinical K. pneumoniae isolates used. Abbreviations: TZP, piperacillin-tazobactam; ATM, aztreonam; CRO, ceftriaxone; FEP, cefepime; ETP, ertapenem; IPM, imipenem; CIP, ciprofloxacin; LVX, levofloxacin; GEN, gentamicin; TOB, tobramycin; R, resistance. MDR strains are marked in red. Download Table S3, DOCX file, 0.02 MB. Copyright © 2023 Huang et al. 2023 Huang et al. https://creativecommons.org/licenses/by/4.0/ This content is distributed under the terms of the Creative Commons Attribution 4.0 International license.

10.1128/msphere.00549-22.7 TABLE S4 MIC values against the five clinical P. aeruginosa isolates used. Abbreviations: TZP, piperacillin-tazobactam; ATM, aztreonam; FEP, cefepime; IPM, imipenem; CIP, ciprofloxacin; LVX, levofloxacin; GEN, gentamicin; TOB, tobramycin; AMK, amikacin; R, resistance. MDR strains are marked in red. Download Table S4, DOCX file, 0.02 MB. Copyright © 2023 Huang et al. 2023 Huang et al. https://creativecommons.org/licenses/by/4.0/ This content is distributed under the terms of the Creative Commons Attribution 4.0 International license.

10.1128/msphere.00549-22.8 TABLE S5 MIC values or Kirby-Bauer values against the five clinical E. cloacae isolates used. Abbreviations: CFZ, cefazolin; CTT, cefotetan; ATM, aztreonam; CRO, ceftriaxone; ETP, ertapenem; IPM, imipenem; CIP, ciprofloxacin; LVX, levofloxacin; GEN, gentamicin; TOB, tobramycin; AMK, amikacin; NIT, nitrofurantoin. MDR strains are marked in red; R, resistance. Kirby-Bauer values are marked in blue. Download Table S5, DOCX file, 0.02 MB. Copyright © 2023 Huang et al. 2023 Huang et al. https://creativecommons.org/licenses/by/4.0/ This content is distributed under the terms of the Creative Commons Attribution 4.0 International license.

10.1128/msphere.00549-22.9 TABLE S6 The timing of blood collection and corresponding injections of Thymol_Au NPs in continuous detection of WBC and NEUT levels. Download Table S6, DOCX file, 0.02 MB. Copyright © 2023 Huang et al. 2023 Huang et al. https://creativecommons.org/licenses/by/4.0/ This content is distributed under the terms of the Creative Commons Attribution 4.0 International license.

### Conclusion.

In summary, the incorporation of natural phenols into Au NPs effectively enhanced their antimicrobial activities. In this study, Thymol_Au NPs were successfully synthesized and showed higher antibacterial activity than last-resort antibiotics against last-resort-antibiotic-resistant bacteria, including TGC-R, COL-R, and CZA-R strains *in vitro* and *in vivo*. The antibacterial mechanism of the Thymol_Au NPs was, at least in part, related to their ability to increase the permeability of the bacterial cell membrane. At bactericidal concentrations, Thymol_Au NPs showed no significant toxicity in almost all tested strains. The changes in WBC, RET (%), and SOD activity in Thymol_Au NP-treated mice warrant attention. In the future, we will focus on how Thymol_Au NPs influence metabolism, transcription, and translation in bacteria to clarify the underlying antibacterial mechanism. We will also explore the clinical application of Thymol_Au NPs in preclinical studies and clinical trials. Au NPs can thus be used as a universal platform that can be adapted to other types of reductive drugs and are effective for mitigating the problem of antibiotic resistance in bacterial pathogens.

## MATERIALS AND METHODS

### Chemicals and reagents.

The main materials used in this study and their manufacturers are listed in Table S7.

10.1128/msphere.00549-22.10 TABLE S7 The main materials used in the study and their manufacturers. Download Table S7, DOCX file, 0.02 MB. Copyright © 2023 Huang et al. 2023 Huang et al. https://creativecommons.org/licenses/by/4.0/ This content is distributed under the terms of the Creative Commons Attribution 4.0 International license.

### Bacterial isolates.

Thirty-one nonduplicate clinical Gram-negative bacterial strains resistant to last-resort antibiotics, including 9 TGC-R, 10 COL-R, and 12 CZA-R strains, were isolated at the First Affiliated Hospital of Wenzhou Medical University. All isolates were named by the First Affiliated Hospital of Wenzhou Medical University, specifically, “DC*XXXX*” for E. coli, “FK*XXXX*” for K. pneumoniae, “CG*XXXX*” for E. cloacae, “TL*XXXX*” for P. aeruginosa, and “BM*XXXX*” for A. baumannii. All isolates were identified using matrix-assisted laser desorption ionization–time of flight mass spectrometry (MALDI-TOF MS; bioMérieux, Lyon, France) per the manufacturer’s instructions. The Vitek 2 compact system (bioMérieux, Lyon, France) was used to determine the MIC values of the most common antibiotics. Kirby-Bauer tests were also used to determine the sensitivities of the strains to antibiotics. The National Center for Clinical Laboratory provided the quality control strain E. coli ATCC 25922 for use in testing.

### Preparation and characterization of different Au NPs.

Au NPs were prepared as previously described, with some modifications (35). In brief, natural phenols (including thymol, plumbagin, naringenin, and kaempferol, 0.05 mmol), Tween 80 (50 mg), and triethylamine (50 μL) were dissolved in 10 mL ice-cold water with ultrasonication for 20 min to obtain a mixture. HAuCl_4_·3H_2_O (0.05 mmol, 400 μL) was added dropwise to the mixture with vigorous stirring (1,000 rpm) in an ice-cold water bath. The mixture was stirred continuously for a further 2 h. The formation of Au NPs was indicated by the development of a purple color. To remove unreacted compounds, the Au NPs were dialyzed against double-distilled water for 24 h, and the mixture was sterilized by filtering through a 0.22-μm filter. The charge and dispersibility of the nanoparticles were assessed using a nanoparticle size and zeta potential analyzer (DLS) (Malvern Zetasizer Nano ZS90; Malvern, UK). A multifunctional microplate reader (BioTek Synergy Neo_2_; BioTek, USA) was used to measure the UV-visible (UV-vis) absorption of different Au NPs. The morphologies of different Au NPs were characterized by TEM (JEOL JEMF200; JEOL, Japan). The concentrations of different Au NPs were determined by measuring the elemental gold using ICP-OES (Thermo Fisher iCAP PRO; Thermo Fisher, USA).

### Antibacterial activity *in vitro*.

The MIC values of TGC, COL, and CZA were measured according to the *Performance Standards for Antimicrobial Susceptibility Testing* (32nd edition) published by the Clinical and Laboratory Standards Institute. The MIC values of Thymol_Au NPs, Plumbagin_Au NPs, Naringenin_Au NPs, Kaempferol_Au NPs, thymol, and unmodified Au NPs were measured as previously described, with some modifications (35). An overnight-cultivated isolated bacterial colony was diluted to a McFarland standard of 0.5 in NS and then diluted once more to a 1:100 concentration in Luria-Bertani (LB) medium. The prepared bacterial suspension was diluted with various concentrations of Thymol_Au NPs, Plumbagin_Au NPs, Naringenin_Au NPs, Kaempferol_Au NPs, thymol, and unmodified Au NPs to a final volume of 100 μL. After incubation for 16 to 20 h at 37°C, the growth of the bacteria was assessed by visual inspection. The MIC was defined as the lowest concentration that prevented the tested isolate from exhibiting observable growth. For TEM/EDS analysis, Thymol_Au NP-treated bacteria were collected at the bottom of the tube followed by the slow addition of precooled (4°C) 2.5% glutaraldehyde. The samples were stored overnight at 4°C, after which the glutaraldehyde was removed. The sample was washed three times with PBS and then fixed with 1% osmic acid for 1 to 2 h. Later, the sample was dehydrated with an ethanol gradient (30, 50, 70, 80, 90, and 95%) in sequence for 15 min at each concentration, followed by incubation with 100% ethanol for 20 min. The sample was then incubated with pure acetone for 20 min, followed by treatment with a mixed solution of embedding medium and acetone (1/1 [vol/vol]) for 1 h. The sample was then treated with different concentrations (3/1 [vol/vol]) of embedding medium and acetone for 3 h and incubated with embedding medium overnight. The samples were then embedded by heating overnight at 70°C and sectioned on an ultramicrotome to obtain 70- to 90-nm sections, which were then stained with lead citrate solution and 50% uranyl acetate-saturated ethanol solution for 5 to 10 min and observed under TEM after drying. For live/dead bacterial cell viability assay, bacteria were treated with Thymol_Au NPs at 37°C for 4 h and stained with SYTO 9 (0.5 μM) and PI (3 μM), per the manufacturer’s instructions. The samples were observed under a confocal microscope (Nikon A1R-SIM-Storm; Nikon, Japan) at excitation wavelengths of 488 and 561 nm and emission wavelengths of 530 (green) and 617 (red) nm. For the intracellular protein leakage assay, bacterial suspensions (10^7^ CFU/mL) were mixed with different concentrations of Thymol_Au NPs (25, 12.5, 6.25, and 3.125 μg/mL) and incubated at 37°C for 6 h. The suspensions were centrifuged at 5,000 rpm for 10 min at 4°C. A bicinchoninic acid (BCA) protein detection kit was used to quantify intracellular protein leakage in supernatants per the manufacturer’s instructions. For SEM experiments, silicon wafers (5 by 5 mm) were placed in 24-well plates to provide a biofilm-forming surface. To achieve the desired cell density of 10^6^ CFU/mL, 10 μL of fresh cell suspension was added to 990 μL of LB broth containing PBS or Thymol_Au NPs. The cultures were incubated for 24 h at 37°C. Then, the silicon wafers were washed three times with PBS, fixed with 2.5% glutaraldehyde for 30 min, and dehydrated in an ethanol gradient (30, 50, 70, 80, 90, 95, and 100%) for 5 min at each concentration. The final samples were air dried, gold sprayed, and observed by SEM (Hitachi SU8010; Hitachi, Japan).

### Antibacterial activity *in vivo*.

Male ICR mice (Vital River, Beijing, China) (6 weeks old and specific pathogen free) were used in this experiment. The mice were maintained in accordance with the National Standards for Laboratory Animals of China (GB 14925-2010). All animal studies were approved by the Zhejiang Association for Science and Technology (identifier [ID] SYXK [Zhejiang] 2018-0017) and were carried out in compliance with the Wenzhou Laboratory Animal Welfare and Ethics standards. FK6768 was randomly selected for the single-dose treatment model. Cyclophosphamide (150 mg/kg of body weight) was injected into the mouse abdominal cavity for 3 days to establish an immune deficiency model. Mice were randomly divided into three groups of 10 and injected intraperitoneally with 200 μL bacterial suspension (10^8^ CFU/mL). After 2 h, PBS, TGC (10 mg/kg), or Thymol_Au NPs (10 mg/kg) were injected into the abdominal cavity. The therapeutic dose of Thymol_Au NPs was in line with the dose of aminophenol-decorated gold nanoparticles used by Wang et al. (35). Body weight measurements were carried out at 0, 6, 12, 24, 36, 48, 60, and 72 h. In the continuous treatment experiment, FK6768, TL3008, and TL3077 were randomly chosen. After 3 days of immunosuppression, a bacterial suspension was injected into the abdominal cavity. Then, PBS, TGC (10 mg/kg) for FK6768-infected mice, COL (10 mg/kg) for TL3008-infected mice, CZA (10/4 mg/kg) for TL3077-infected mice, or Thymol_Au NPs (10 mg/kg) were injected at 0, 12, 24, and 36 h. Body weight measurements were carried out at 0, 6, 12, 18, 24, 36, 48, 60, and 72 h. Different organs (heart, liver, spleen, lung, and kidney) were homogenized in PBS and spread on LB agar plates to observe the bacterial burden.

### Biocompatibility *in vitro*.

For the cell viability assay, 100 μL of Huh-7 and RAW 264.7 cell suspensions containing 2,000 cells was seeded into each well of a 96-well plate. Later, 10 μL of test agent (PBS or different concentrations of Thymol_Au NPs) was added to the medium and the plate was incubated for 12 h. Following incubation, 10 μL of CCK-8 reagent was added to each well and the plate was incubated for a further 1 h at 37°C. The absorbance at 450 nm was measured using a microplate reader. The percentage of cell viability was calculated as follows: cell viability (%) = (absorbance of sample − absorbance of medium)/(absorbance of negative control − absorbance of medium) × 100.

### Biocompatibility *in vivo*.

Healthy mice were randomly divided into five groups of three mice each, namely, the (i) PBS, (ii) TGC, (iii) COL, (iv) CZA, and (v) Thymol_Au NP groups. The dose and frequency of injected solutions were consistent with those used in the continuous treatment model. After 48 h, the mice were sacrificed and the major organs (heart, liver, spleen, lung, and kidney) were collected and fixed in 4% formaldehyde solution. The tissues were then embedded in paraffin and cut into 5-μm sections which were then stained with hematoxylin and eosin (H&E). Blood samples were also collected after 48 h from the orbital plexus for routine blood tests. As for the continuous testing of the WBC and NEUT levels, we measured these values at 0, 12, 24, 36, and 48 h. An animal-specific hematology analyzer (Sysmex XN-1000; Sysmex, Japan) was used for blood analysis. SOD activities and MDA levels were analyzed in sera obtained from the blood samples at 0 and 48 h using the corresponding assay kits.

### Statistical analysis.

Data were expressed as mean ± standard deviation from at least three independent experiments. Statistical significance was determined using an independent two-tailed *t* test and expressed as *P* values of <0.05 (denoted by *), <0.01 (denoted by **), and <0.001 (denoted by ***). GraphPad Prism 9.4 was used for statistical analysis.
