# Supplementary material for: Thymol-Decorated Gold Nanoparticles for Curing Clinical Infections Caused by Bacteria Resistant to Last-Resort Antibiotics
Source: mSphere. 2023 Apr 5;8(3):e00549-22. doi: 10.1128/msphere.00549-22 (PMC10286717; doi:10.1128/msphere.00549-22)
Supplement: TABLE S1 [file msphere.00549-22-s0004.docx]

| **Strains** | **MIC values (μg/ml)** | | | | | | | | | | | | | | | |
| --- | --- | --- | --- | --- | --- | --- | --- | --- | --- | --- | --- | --- | --- | --- | --- | --- |
|  | **AMP** | **SAM** | **TZP** | **CFZ** | **CTT** | **ATM** | **CRO** | **FEP** | **ETP** | **IPM** | **CIP** | **LVX** | **GEN** | **TOB** | **AMK** | **NIT** |
| DC7914 | ≥32^R^ | ≥32/16^R^ | ≥128/4^R^ | ≥64^R^ | ≥64^R^ | ≥64^R^ | ≥64^R^ | ≥64^R^ | ≥8^R^ | ≥16^R^ | ≥4^R^ | ≥8^R^ | ≥16^R^ | ≥16^R^ | ≥64^R^ | 64^R^ |
| DC3599 | ≥32^R^ | ≥32/16^R^ | ≤4/4 | ≥64^R^ | ≤4 | ≥64^R^ | ≥64^R^ | 8 | ≤0.5 | ≤1 | ≥4^R^ | ≥8^R^ | ≥16^R^ | 8 | ≤2 | ≤16 |
| DC5286 | ≥32^R^ | ≥32/16^R^ | 64/4 | ≥64^R^ | ≤4 | 8 | ≥64^R^ | 4 | ≤0.5 | ≤1 | ≥4^R^ | 4^R^ | ≤1 | ≤1 | ≤2 | ≤16 |
| DC8439 | ≥32^R^ | ≥32/16^R^ | ≥128/4^R^ | ≥64^R^ | ≥64^R^ | ≥64^R^ | ≥64^R^ | ≥64^R^ | ≥8^R^ | ≥16^R^ | ≥4^R^ | ≥8^R^ | ≥16^R^ | 8 | ≤2 | ≤16 |
| DC7956 | ≥32^R^ | ≥32/16^R^ | ≥128/4^R^ | ≥64^R^ | ≥64^R^ | ≥64^R^ | ≥64^R^ | ≥64^R^ | ≥8^R^ | ≥16^R^ | ≥4^R^ | ≥8^R^ | ≥16^R^ | ≥16^R^ | ≥64^R^ | 64^R^ |
