# Supplementary figures and images for: Thymol-Decorated Gold Nanoparticles for Curing Clinical Infections Caused by Bacteria Resistant to Last-Resort Antibiotics
Source: mSphere. 2023 Apr 5;8(3):e00549-22. doi: 10.1128/msphere.00549-22 (PMC10286717; doi:10.1128/msphere.00549-22)

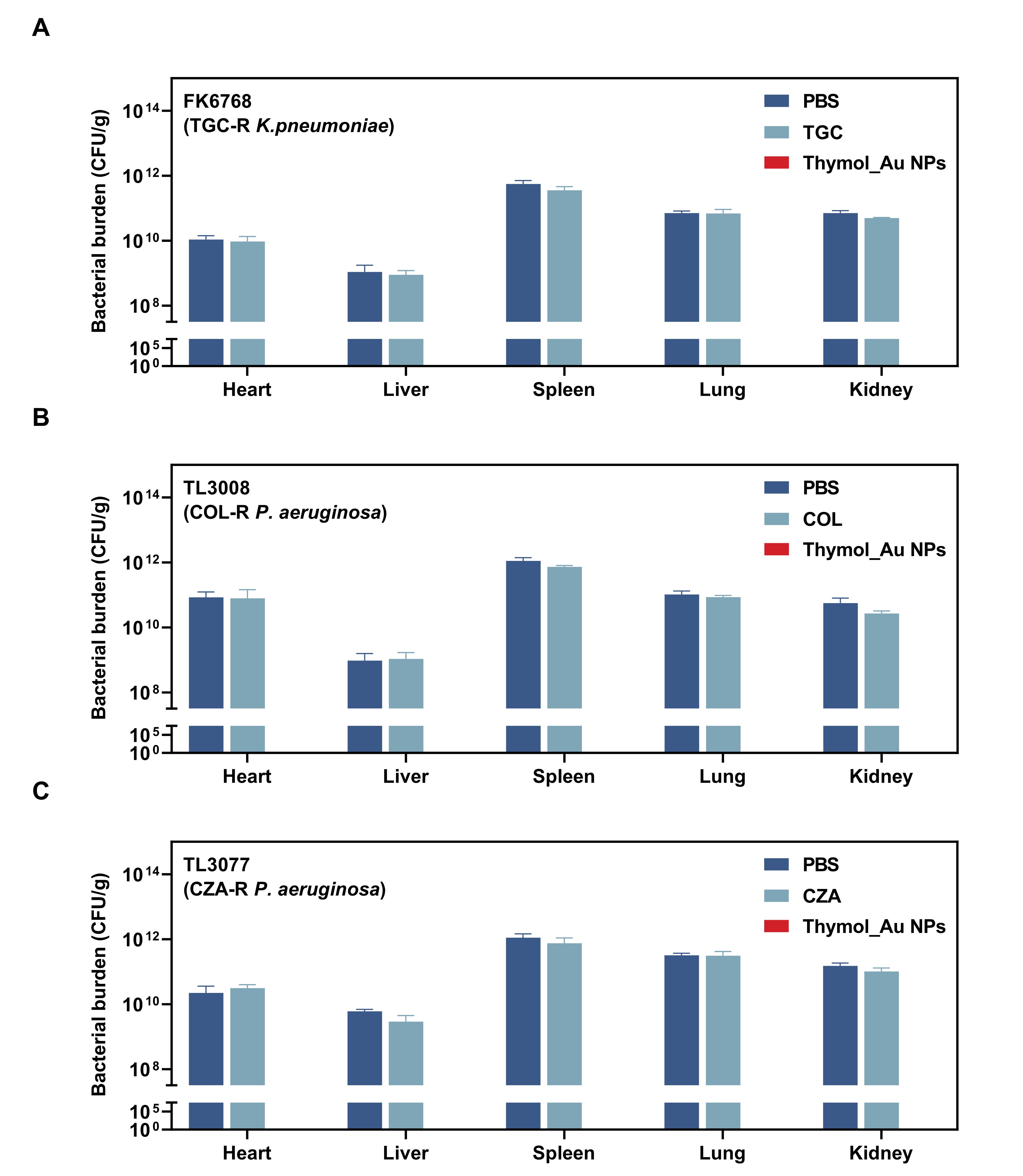

Supplement: FIG S1 [file msphere.00549-22-s0001.tif]

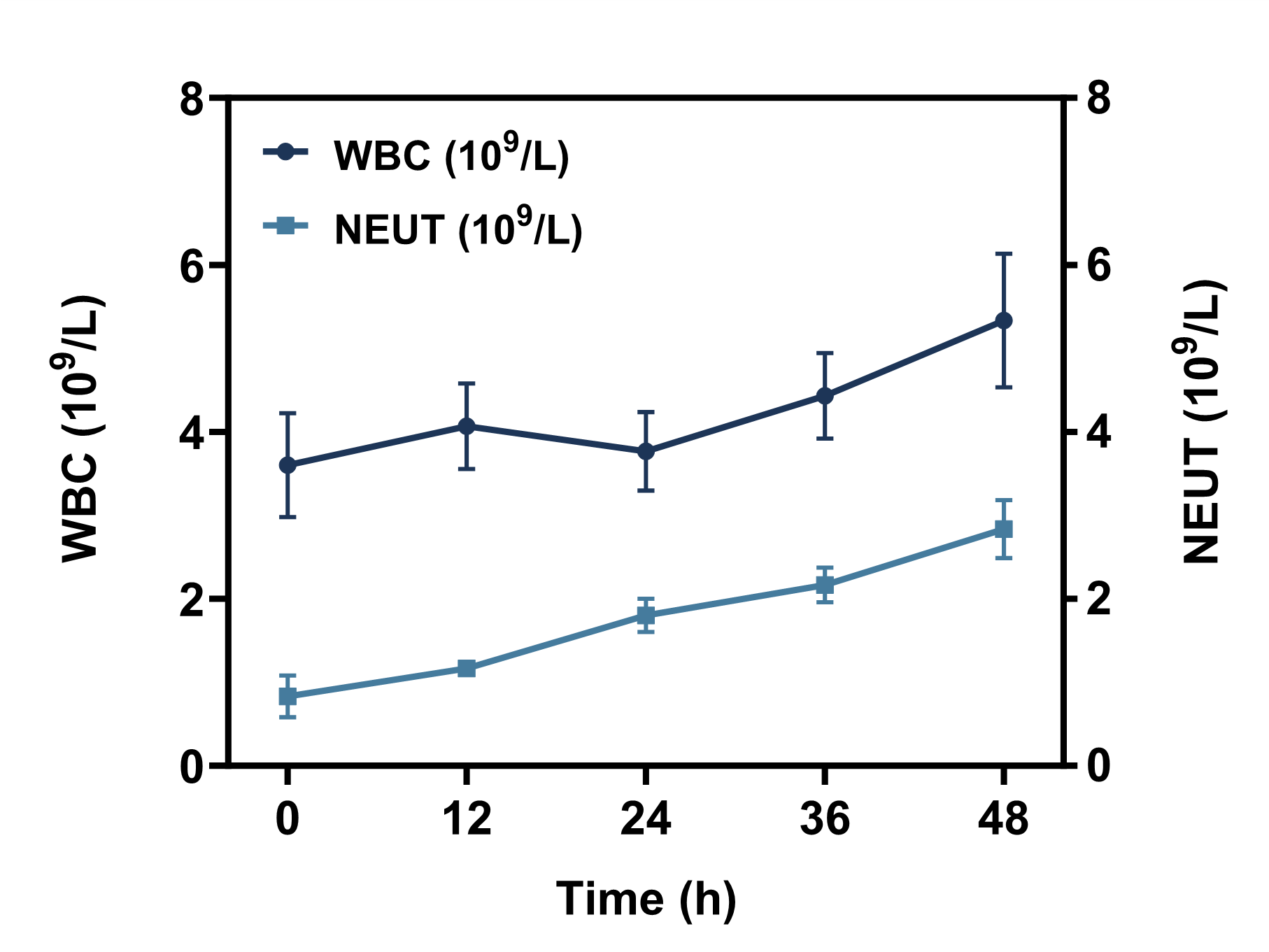

Supplement: FIG S2 [file msphere.00549-22-s0002.tif]

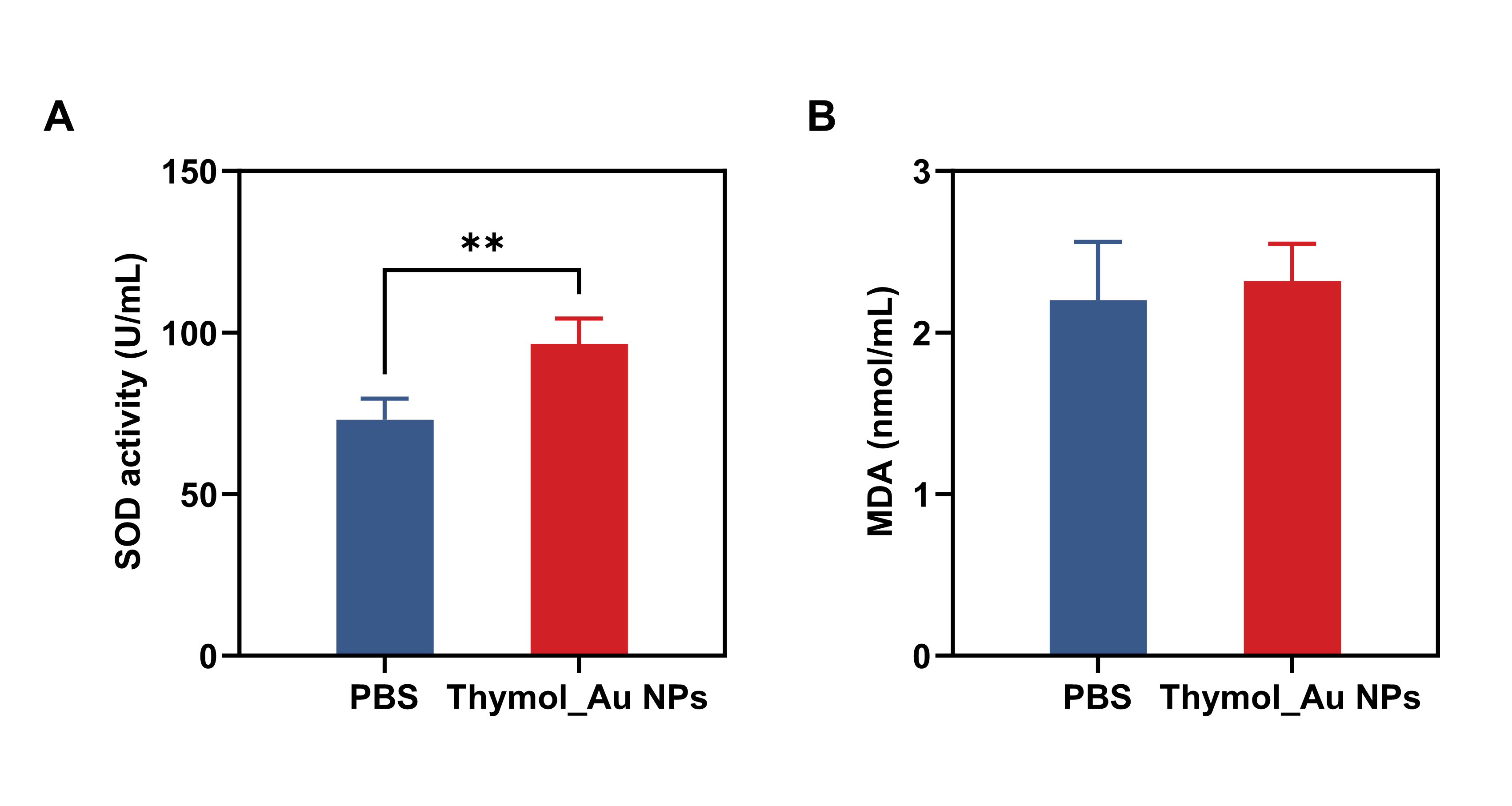

Supplement: FIG S3 [file msphere.00549-22-s0003.tif]
