# Supplementary material for: Thymol-Decorated Gold Nanoparticles for Curing Clinical Infections Caused by Bacteria Resistant to Last-Resort Antibiotics
Source: mSphere. 2023 Apr 5;8(3):e00549-22. doi: 10.1128/msphere.00549-22 (PMC10286717; doi:10.1128/msphere.00549-22)
Supplement: TABLE S2 [file msphere.00549-22-s0005.docx]

| **Strains** | **MIC values (μg/ml)** | | | | | |
| --- | --- | --- | --- | --- | --- | --- |
|  | **SAM** | **CRO** | **FEP** | **IPM** | **CIP** | **LVX** |
| BM7580 | ≥32/16^R^ | ≥64^R^ | ≥64^R^ | ≥16^R^ | ≥4^R^ | 4 |
| BM7438 | ≥32/16^R^ | ≥64^R^ | ≥64^R^ | ≥16^R^ | ≥4^R^ | ≥8^R^ |
| BM1595 | ≥32/16^R^ | ≥64^R^ | ≥64^R^ | ≥16^R^ | ≥4^R^ | 4 |
| BM7389 | 16/8 | ≥64^R^ | 32 | ≥16^R^ | ≥4^R^ | ≥8^R^ |
| BM7594 | ≥32/16^R^ | ≥64^R^ | ≥64^R^ | ≥16^R^ | ≥4^R^ | 4 |
| BM7404 | ≥32/16^R^ | ≥64^R^ | ≥64^R^ | ≥16^R^ | ≥4^R^ | ≥8^R^ |
| BM5333 | 16/8 | 32 | ≥64^R^ | ≥16^R^ | ≥4^R^ | ≥8^R^ |
| BM7338 | ≥32/16^R^ | ≥64^R^ | ≥64^R^ | ≥16^R^ | ≥4^R^ | ≥8^R^ |
| BM7481 | ≥32/16^R^ | ≥64^R^ | ≥64^R^ | ≥16^R^ | ≥4^R^ | ≥8^R^ |
| BM2431 | ≥32/16^R^ | ≥64^R^ | ≥64^R^ | ≥16^R^ | ≥4^R^ | ≥8^R^ |
