# Supplementary material for: Thymol-Decorated Gold Nanoparticles for Curing Clinical Infections Caused by Bacteria Resistant to Last-Resort Antibiotics
Source: mSphere. 2023 Apr 5;8(3):e00549-22. doi: 10.1128/msphere.00549-22 (PMC10286717; doi:10.1128/msphere.00549-22)
Supplement: TABLE S3 [file msphere.00549-22-s0006.docx]

| **Strains** | **MIC values (μg/ml)** | | | | | | | | | |
| --- | --- | --- | --- | --- | --- | --- | --- | --- | --- | --- |
|  | **TZP** | **ATM** | **CRO** | **FEP** | **ETP** | **IPM** | **CIP** | **LVX** | **GEN** | **TOB** |
| FK3810 | ≥128/4^R^ | ≥64^R^ | ≥64^R^ | ≥64^R^ | ≥8^R^ | ≥16^R^ | ≥4^R^ | ≥8^R^ | ≥16^R^ | ≥16^R^ |
| FK8966 | ≥128/4^R^ | ≥64^R^ | ≥64^R^ | ≥64^R^ | ≥8^R^ | ≥16^R^ | ≥4^R^ | 1 | ≥16^R^ | 8 |
| FK9102 | ≥128/4^R^ | ≥64^R^ | ≥64^R^ | ≥64^R^ | ≥8^R^ | ≥16^R^ | ≥4^R^ | ≥8^R^ | ≤1 | 4 |
| FK6768 | ≥128/4^R^ | ≥64^R^ | ≥64^R^ | ≥64^R^ | ≥8^R^ | ≥16^R^ | ≥4^R^ | ≥8^R^ | ≤1 | ≤1 |
| FK9283 | ≤4/4 | ≤1 | ≤1 | ≤1 | ≤0.5 | ≤1 | ≤0.25 | ≤0.25 | ≤1 | ≤1 |
| FK1913 | ≥128/4^R^ | ≥64^R^ | ≥64^R^ | 8 | ≤0.5 | ≤1 | ≥4^R^ | ≥8^R^ | ≥16^R^ | ≥16^R^ |
