# Supplementary material for: Thymol-Decorated Gold Nanoparticles for Curing Clinical Infections Caused by Bacteria Resistant to Last-Resort Antibiotics
Source: mSphere. 2023 Apr 5;8(3):e00549-22. doi: 10.1128/msphere.00549-22 (PMC10286717; doi:10.1128/msphere.00549-22)
Supplement: TABLE S4 [file msphere.00549-22-s0007.docx]

| **Strains** | **MIC values (μg/ml)** | | | | | | | | |
| --- | --- | --- | --- | --- | --- | --- | --- | --- | --- |
|  | **TZP** | **ATM** | **FEP** | **IPM** | **CIP** | **LVX** | **GEN** | **TOB** | **AMK** |
| TL3008 | 8/4 | 4 | 8 | ≥16^R^ | 0.5 | 1 | 4 | ≤1 | 8 |
| TL3086 | 8/4 | 16^R^ | 8 | ≥16^R^ | ≤0.25 | 1 | 8 | ≤1 | 8 |
| TL3077 | 8/4 | 4 | 2 | 4 | 0.5 | 1 | 2 | ≤1 | ≤2 |
| TL2918 | 64/4^R^ | ≥64^R^ | ≥64^R^ | ≥16^R^ | 1 | 2 | ≤1 | ≤1 | 4 |
| TL2964 | 64/4^R^ | 16^R^ | ≥64^R^ | ≥16^R^ | ≥4^R^ | ≥8^R^ | ≥16^R^ | ≥16^R^ | ≥64^R^ |
