# Supplementary material for: Thymol-Decorated Gold Nanoparticles for Curing Clinical Infections Caused by Bacteria Resistant to Last-Resort Antibiotics
Source: mSphere. 2023 Apr 5;8(3):e00549-22. doi: 10.1128/msphere.00549-22 (PMC10286717; doi:10.1128/msphere.00549-22)
Supplement: TABLE S5 [file msphere.00549-22-s0008.docx]

| **Strains** | **MIC values (μg/ml) or Kirby–Bauer values (mm)** | | | | | | | | | | | |
| --- | --- | --- | --- | --- | --- | --- | --- | --- | --- | --- | --- | --- |
|  | **CFZ** | **CTT** | **ATM** | **CRO** | **ETP** | **IPM** | **CIP** | **LVX** | **GEN** | **TOB** | **AMK** | **NIT** |
| CG648 | ≥64^R^ | ≤4 | 16^R^ | 16^R^ | 4^R^ | ≥16^R^ | ≤0.25 | ≤0.25 | ≤1 | ≤1 | ≤2 | 32 |
| CG1400 | ≥64^R^ | ≥64^R^ | ≥64^R^ | ≥64^R^ | ≥8^R^ | ≤1 | ≥4^R^ | ≥8^R^ | ≥16^R^ | ≥16^R^ | 4 | 64 |
| CG741 | ≥64^R^ | ≥64^R^ | ≤1 | 4 | 4^R^ | ≤1 | ≥4^R^ | ≥8^R^ | ≤1 | 8 | ≤2 | 64 |
| CG1593 | ≥64^R^ | ≥64^R^ | 32^R^ | ≥64^R^ | 16^R^ | 18^R^ | 0.5 | 1 | ≤1 | 8 | ≤2 | ≤16 |
| CG1257 | ≥64^R^ | ≥64^R^ | 28 | ≥64^R^ | 16^R^ | 17^R^ | ≤0.25 | 1 | 4 | 8 | ≤2 | 64 |
