# Supplementary material for: Thymol-Decorated Gold Nanoparticles for Curing Clinical Infections Caused by Bacteria Resistant to Last-Resort Antibiotics
Source: mSphere. 2023 Apr 5;8(3):e00549-22. doi: 10.1128/msphere.00549-22 (PMC10286717; doi:10.1128/msphere.00549-22)
Supplement: TABLE S6 [file msphere.00549-22-s0009.docx]

| **Blood collection** | **Injection of Thymol Au NPs** |
| --- | --- |
| 0 h | No injection |
| 12 h | 0 h |
| 24 h | 0, 12 h |
| 36 h | 0, 12, 24 h |
| 48 h | 0, 12, 24, 36 h |
