# Supplementary material for: Thymol-Decorated Gold Nanoparticles for Curing Clinical Infections Caused by Bacteria Resistant to Last-Resort Antibiotics
Source: mSphere. 2023 Apr 5;8(3):e00549-22. doi: 10.1128/msphere.00549-22 (PMC10286717; doi:10.1128/msphere.00549-22)
Supplement: TABLE S7 [file msphere.00549-22-s0010.docx]

| **Material** | **Manufacturer** |
| --- | --- |
| HAuCl_4_ | Macklin (Shanghai, China) |
| Tween 80 | Macklin (Shanghai, China) |
| Triethylamine | Wenzhou Jinshan Chemical Reagent Instrument Co., Ltd (Zhejiang, China) |
| Thymol | Sigma-Aldrich (America) |
| Plumbagin | MedChemExpress (Shanghai, China) |
| Naringenin | MedChemExpress (Shanghai, China) |
| Kaempferol | Shanghai Yuanye Bio-Technology Co., Ltd (Shanghai, China) |
| CAZ | Solarbio (Beijing, China) |
| AVI | Wenzhou Kangtai Biological Technology Co., Ltd (Zhejiang, China) |
| TGC | Wenzhou Kangtai Biological Technology Co., Ltd (Zhejiang, China) |
| COL | Wenzhou Kangtai Biological Technology Co., Ltd (Zhejiang, China) |
| NaBH_4_ | Macklin (Shanghai, China) |
| CCK-8 | MedChemExpress (Shanghai, China) |
| BCA protein assay kit | New Cell & Molecular Biotech Co., Ltd (Jiangsu, China) |
| Live/dead bacLight bacterial viability kit | Invitrogen (America) |
| Cyclophosphamide | Shanghai Yuanye Bio-Technology Co., Ltd (Shanghai, China) |
| 2.5% glutaraldehyde | Servicebio (Hubei, China) |
| Total superoxide dismutase assay kit | Nanjing Jiancheng Bioengineering Institute (Jiangsu, China) |
| Malondialdehyde assay kit | Nanjing Jiancheng Bioengineering Institute (Jiangsu, China) |
